# Comparison of mouse mammary gland imaging techniques and applications: Reflectance confocal microscopy, GFP Imaging, and ultrasound

**DOI:** 10.1186/1471-2407-8-21

**Published:** 2008-01-23

**Authors:** Maddalena T Tilli, Angela R Parrish, Ion Cotarla, Laundette P Jones, Michael D Johnson, Priscilla A Furth

**Affiliations:** 1 Lombardi Comprehensive Cancer Center, Georgetown University, Washington, DC 20057, USA; 2 (current address) The Jack H. Skirball Center for Chemical Biology & Proteomics, The Salk Institute for Biological Studies, 10010 N. Torrey Pines Road, La Jolla, CA 9203, USA; 3 (current address) University of Maryland Medical School, Department of Pharmacology and Experimental Therapeutics, Baltimore, MD 21201, USA

## Abstract

**Background:**

Genetically engineered mouse models of mammary gland cancer enable the *in vivo *study of molecular mechanisms and signaling during development and cancer pathophysiology. However, traditional whole mount and histological imaging modalities are only applicable to non-viable tissue.

**Methods:**

We evaluated three techniques that can be quickly applied to living tissue for imaging normal and cancerous mammary gland: reflectance confocal microscopy, green fluorescent protein imaging, and ultrasound imaging.

**Results:**

In the current study, reflectance confocal imaging offered the highest resolution and was used to optically section mammary ductal structures in the whole mammary gland. Glands remained viable in mammary gland whole organ culture when 1% acetic acid was used as a contrast agent. Our application of using green fluorescent protein expressing transgenic mice in our study allowed for whole mammary gland ductal structures imaging and enabled straightforward serial imaging of mammary gland ducts in whole organ culture to visualize the growth and differentiation process. Ultrasound imaging showed the lowest resolution. However, ultrasound was able to detect mammary preneoplastic lesions 0.2 mm in size and was used to follow cancer growth with serial imaging in living mice.

**Conclusion:**

In conclusion, each technique enabled serial imaging of living mammary tissue and visualization of growth and development, quickly and with minimal tissue preparation. The use of the higher resolution reflectance confocal and green fluorescent protein imaging techniques and lower resolution ultrasound were complementary.

## Background

Transgenic mouse models have been developed to recapitulate the complex effects of genes known to be involved in human breast cancer. These models can help to elucidate the mechanism of action of these genes during carcinogenesis, as well as their impact on normal mammary gland development. Imaging methods for mouse models of normal and cancerous mammary glands are in the developing stages and can help in the search for better ways to diagnosis human breast cancer earlier [1].

In order to study early phenotypic effects of gene over-expression or lack of expression on mammary gland development and cancer traditional methods require that tissue is harvested from the animal and subjected to histological techniques to detect morphologically aberrant growth. These invasive procedures preclude further examination of the effects of these genetic changes on the process of carcinogenesis. Later, once the tumor becomes palpable, the size of the developing tumor can be measured and followed *in vivo *over time to determine proliferative capacity. However, no information about initiation and progression can be gathered; only at the end of the experiment [2] can information about the morphology or gene expression profile of the developing tumor be obtained.

The implementation of *in vivo *imaging modalities to study normal mammary gland growth and disease progression has greatly improved the utility of these models, allowing the study of mammary differentiation or disease process, not simply the final effect [3]. Biochemical and morphologic changes associated with early cancer change the optical properties of tissue, especially the absorption, scattering, and fluorescence, allowing the detection of these early carcinogenic effects with optical spectroscopy techniques [4]. Imaging can allow researchers to, with minimal invasiveness, detect and follow abnormalities in ductal development during mammary differentiation in the same living animal. In cancer studies, imaging can detect undissected preneoplastic lesions and follow the behavior of these cancer cells, interactions with their stromal environment during the development of a tumor, angiogenesis, and metastatic disease. This can all be studied over time in the context of the cancer cells own physiological environment with an intact blood supply and interaction with surrounding tissues in 3-D and in real-time [1,5,6]. Imaging regimens can also be adapted to evaluate efficacy and response of a cancer to prevention and therapeutic interventions [7,8] and to detect the presence of chemoresistance [9]. Serial minimally invasive imaging of mice reduces the number of mice needed per experiment or in preclinical drug development since multiple time points can be observed in the same animal [8]. The imaging modalities reflectance confocal microscopy (RCM), green fluorescent protein (GFP) imaging, and ultrasound imaging were utilized in this paper to image mammary glands and mammary tumors.

RCM provides real-time minimally invasive 3-D sectioning of *in vivo *(living) or *ex vivo *(newly biopsied) individual cells and tissues using variations in the optical properties of the natural backscattering of light from different cellular and subcellular structures without the use of labeling cells fluorescently or otherwise [10,11]. Optical techniques such as RCM have demonstrated high sensitivity for detecting cancer in their natural environment without using ionizing radiation [12] and without time-consuming and potentially destructive fixation and staining, both of which may introduce artifacts and damage tissue [13]. Tissue studied with RCM is treated with acetic acid, which induces DNA condensation providing increased reflectance to contrast nuclear versus cytoplasmic structure. We have shown that tissue treated with acetic acid can then be subjected to histological and immunohistological analyses without detrimental effects on the tissue [14], facilitating further study into signaling pathways which may be active in the imaged structure [15]. RCM has been performed on biopsy specimens to assess tumor margins [16] and to identify precancerous lesions in human breast core needle biopsies [14], the cervix [17], and skin [18].

Fluorescent protein labeling and epi-illumination spectroscopy microscopy are very powerful tools to follow primary tumor growth and metastasis with fluorophores *in vivo *and in real time [19]. Transgenic GFP optical imaging is one type of fluorescent protein label imaging and involves the detection of reporter transgene expression, namely a genetically encoded fluorescent protein, which is utilized to image cells within living tissue [3,20]. The specimen, often exposed surgically, is illuminated with blue light (488 nm excitation wavelength) which is absorbed by green fluorescent protein, a protein originally from the jellyfish *Aequorea Victoria *[6]. GFP then emits green light (509 nm peak shifted emission wavelength) which is collected by CCD cameras [21]. GFP imaging can be used as a cell marker in both the living animal and in tissue culture and does not require a substrate for visualization [22].

GFP transfected tissue culture cells and GFP transgenic mice have been used to monitor real time tumor growth and for mechanistic studies [23,24], evaluate the efficacy of therapy in a tumor xenograft model with metastasis [25], monitor specificity of *in vivo *gene therapy studies [26], mark and sort potential mammary stem cells [27], and examine mammary epithelial tumor cell behavior in metastasis [28]. In addition to monitoring mammary gland development on the whole at the ductal morphology level as in our current study, GFP can be used to image single cells. This high resolution GFP imaging of cells *in vivo *has been combined in a dual labeling approach with red fluorescent protein (RFP) to monitor tumor-stroma interactions and drug response of cancer and stromal cells [29,30].

Ultrasound imaging involves exposing tissues to high-frequency ultrasound waves (20–60 MHz in animals; 2–10 MHz in humans) by placing a transducer (which contains crystals that vibrate when exposed to small electrical currents and produce sound waves) on the skin and then detecting the ultrasound reflections from internal organs under investigation [6,31,32]. This non-invasive technique produces a dynamic real-time image of the tissue from which structural and functional information can be obtained because sound waves travel though soft tissue based on the acoustic impedance of each tissue, which is a function of the tissue density [31]. When two tissues with different densities are next to each other, a mismatch in the acoustic impedance causes sound waves to be reflected relative to the degree of mismatch; a greater acoustic impedance mismatch leads to a greater reflected pressure magnitude or intensity and is seen as a brighter image [31].

Ultrasound is a rapid non-radiation method that has been used to detect cystic masses [33] and superficial tumors [34], differentiate between fibroadenomas and carcinomas in animal models [35], noninvasively track liver metastases growth and evaluate potential therapy in liver metastasis models [36], measure blood flow by Doppler [37,38], guide biopsy of a palpable breast mass [38], and guide injections into target organs [39].

In the present study, we use RCM, GFP, and ultrasound to visualize mammary gland and mammary tumor characteristics *in vivo*. We show that RCM can be used to study mammary development in an *ex vivo *whole organ culture setting with good resolution using a lower concentration of acetic acid. We show that GFP expression can be used to visualize mammary gland ducts, mammary tumor, and tumor vasculature, to follow lobuloaveolar development in an *ex vivo *whole organ culture experiment, and can be used to follow development of transplanted mammary glands. We show that ultrasound imaging can be used to visualize normal mammary gland, hyperplastic areas of preneoplasia, to follow tumor progression and liver metastases, and can be used to distinguish between mammary tumor and enlarged lymph node. In conclusion, we show that these modalities are, individually and in combination, useful in studying normal and carcinogenic biological processes in the mouse mammary gland longitudinally and with minimal invasiveness.

## Methods

### Mouse Models, Mammary Gland Whole Mounts, and Hematoxylin and Eosin Sections

Mammary glands from wild-type C57Bl/6 female mice, female mice from a model of Estrogen Receptor alpha (ERα) driven mammary cancer (tTA/TAg/ERα mice) [40], and female mice from a WAP-TAg mammary cancer model [41] were examined by different imaging modalities in this study. In general, after imaging was performed, one #4 mammary gland was fixed in formalin overnight, embedded in paraffin, slices (5 μm) mounted on glass slides, and stained with Hematoxylin and Eosin (H&E). For mammary gland whole mount staining, the other #4 mammary gland was fixed in Carnoy's fixative and stained in Carmine-alum as previously described [42]. Visualization of carmine-alum and H&E stained mammary glands was performed on an Eclipse E800M microscope (Nikon Instruments Inc., Melville, NY, USA). Mammary gland preneoplastic lesions were measured *in situ *upon necropsy to compare with measurements taken with the ultrasound software (n = 7). For the metastasis study, the primary mammary adenocarcinoma was removed at 10 months of age. Two weeks after the tumor was removed mammary glands and liver were imaged with ultrasound to screen for the development of new tumors and liver metastases. The mouse was euthanized at 12 months of age because of difficulty breathing and unresponsiveness, 1 month after the original ultrasound and before a second scheduled ultrasound could take place. The presence of liver, lung and omental metastases were confirmed on necropsy. All procedures involving animals were performed in accordance with current federal (National Institutes of Health Guide for the Care and Use of Laboratory Animals) and University guidelines and were reviewed and approved by the Georgetown University Institutional Animal Use and Care Committee.

### Reflectance Confocal Microscopy

Mammary gland ductal and epithelial cell morphology from non-pregnant wild-type mice were directly imaged at different stages of development by reflectance confocal microscopy using the VivaCell 5000 Reflectance Confocal Microscope (VivaCell-TiBa, Rochester, NY, USA) with a 30× water immersion lens. Mice were euthanized prior to reflectance confocal imaging. Upon necropsy, the #3 mammary glands were injected with a dilute (5%) acetic acid in phosphate buffered saline (PBS) solution as a contrast agent to enhance visualization of the nuclei within cells by promoting condensation of nuclear material [43,44]. The mammary gland was then dissected and spread on the microscope stage above the objective on the Vivacell 5000 and images (originally 500 μm × 500 μm) were taken with the VS2000ui imaging software (version vs006.00.11, Lucid, Inc., Rochester, NY), as described previously [15,45]. After imaging, the mammary glands were fixed in formalin for H&E staining as described above.

### GFP Imaging

Transgenic tTA/TAg/ERα mice were bred to mice carrying the enhanced Green Fluorescent Protein (GFP) transgene under the control of the chicken beta-actin promoter coupled with the cytomegalovirus (CMV) immediate early enhancer (FVB.Cg-Tg(ACTB-EGFP)B5Nagy/J strain from The Jackson Laboratories, Bar Harbor, Maine).

These mice express GFP in all cells of the mouse expressing actin, especially in the skin and mammary glands [20]. Due to the nature of adipocytes having low fluorescence, even though they do express GFP, the mammary ductal tree was easily visualized in contrast to the less bright fat. The Nikon SMZ-1500 EPI-Fluorescence Digital Stereoscope System (Melville, NY) was used to visualize GFP-expressing mammary gland and mammary tumor tissues and pictures were taken with the Metamorph imaging software (Molecular Devices Corp, Sunnydale, CA). Using the Apo 1× objective with the 10× eyepiece, the Nikon SMZ-1500 EPI-Fluorescence Digital Stereoscope System has a field of view between 29.3 mm for the 0.75× zoom and 2.0 mm for the 11.25× zoom.

For the *in vivo *GFP experiment, a 4-week-old GFP mouse was anesthetized and incisions made in the abdominal skin such that the skin could be folded back to allow imaging of the ductal epithelial tree in the #4 mammary gland. After images were acquired as above, the skin was closed with surgical staples and the mouse was allowed to recover. At 8 weeks of age the mouse was euthanized and both #4 mammary glands harvested, placed on glass slides, and imaged by GFP fluorescence.

### Mammary Gland Whole Organ Culture

Mammary gland whole organ culture (WOC) was carried out essentially as previously described [46]. Briefly, 21–24 day old wild-type female mice were anesthetized and subcutaneously implanted in the interscapular area with a 21-day release 0.01 mg 17β-estradiol and 10 mg progesterone (E&P) pellet (Innovative Research of America, Sarasota, Florida) to prime the mammary glands for WOC. After 14 days of priming, the mice were sacrificed and the #4 mammary glands were harvested, placed on a square of cotton mesh and floated in WOC growth phase media, Waymouth's MB 752/1 media (Biosource Biofluids, Rockville, MD) supplemented with Antibiotic-Antimycotic (Gibco/Invitrogen, Grand Island, NY), insulin (5 μg/ml), prolactin (1 μg/ml), aldosterone (0.1 μg/ml), hydrocortisone (0.1 μg/ml) (IPAH). Glands were incubated at 50% oxygen, 5% CO2 in humidified air at 37°C (Heraeus Instruments, Newtown, CT).

A WOC experiment was performed to test viability of the mammary glands after treatment with acetic acid and visualization with RCM. Mammary glands of three-week-old wild-type female mice were primed with an E&P pellet as described above. After 14 days of priming, the mice were sacrificed and mammary glands #3 and #4 were harvested. One gland was immediately placed in IPAH WOC media. Before placing the other mammary glands in WOC, they were treated with PBS, 1, 3, or 5% acetic acid in PBS (3 mammary glands per treatment) and were imaged with RCM. After imaging for no more than 5–10 minutes, the mammary glands were placed in IPAH media as described above. After seven days in culture (post-WOC), the #4 mammary glands were whole mounted and #3 mammary glands were fixed and H&E stained as described above. RCM images from mammary glands treated with PBS or acetic acid pre-WOC were compared with the whole mounts and H&E slides post-WOC.

For the WOC GFP images, the mammary glands from mice expressing GFP alone were treated with IPAH for 10 days. Each day, the mammary glands were visualized with GFP imaging to detect and follow changes and growth in the ductal tree.

### Ultrasound

Mice were anesthetized by inhalation of isoflurane with 1–3% oxygen and ventral hair was removed using a mild depilatory cream. Mice were placed on a thermostatically controlled heating pad to help maintain mouse body temperature. A water based ultrasonic gel was applied between the imaging probe transducer and the mouse skin and the liver, mammary tumors, and all ten mammary glands from a total of 16 wild-type, tTA/TAg/ERα, and WAP-TAg mice were imaged with the Visualsonics Vivo 660 High-Resolution Imaging System for small animal ultrasound (Toronto, Ontario, Canada). Transducers were 55 MHz for mammary gland and 40 MHz for liver imaging. Orientation of the mammary gland on ultrasound was accomplished by visualizing the lymph node, which is less echogenic and appears as a black hole surrounded by the echogenic mammary gland tissue. Mammary gland preneoplastic lesions were measured with the ultrasound software using acquired images in the plane showing the largest cross-sectional area of each lesion. These ultrasound measurements were compared with measurements taken at necropsy (n = 7).

### Mammary Gland Transplantation

Mammary gland transplantation is a useful technique to study the specific effects of hormone or even genetic influences on mammary gland growth, differentiation, and carcinogenesis. Unfortunately, it is not always possible to distinguish whether the transplanted mammary gland grew or if it was actually the host mammary gland. Sometimes the host mammary gland does grow even if the fat pad of the host is cleared of epithelial cells, which should ensure that the host mammary gland cannot grow. In order to definitively determine the mammary gland of origin and therefore a successful versus unsuccessful mammary gland transplantation, mammary glands from GFP expressing mice were transplanted into non-GFP expressing hosts. If the mammary gland that grew expressed GFP, then the mammary gland was from the transplanted mammary gland (GFP positive). If the mammary gland that grew did not express GFP, then it was residual host mammary gland (GFP negative). Both #4 mammary glands from 1 to 2-day-old newborn female and male pups were removed under a dissecting stereomicroscope (Zeiss Stemi SV 11, Germany) and placed in a culture dish containing DMEM:Ham's F-12 (1:1) with 10% fetal calf serum, 2 mM glutamine and 1% penicillin-streptomycin to keep the glands moist and maintain tissue viability before transplantation. The mammary glands were then transplanted into 3- to 4-week-old female nude mice without clearing the mammary fat pad: an incision was made and a pocket was created between the #3 and #4 mammary glands in the nude mouse and both mammary glands from the newborn pups were introduced in the pocket [47]. Post-transplantation, a cohort of the host mice were housed with males to become pregnant. Mice that were not housed with males were euthanized 8 weeks after transplantation. For the studies of pregnant transplanted mammary gland, mice that became pregnant were euthanized during late pregnancy (17–19 days pregnancy). For both cohorts, the transplanted glands were removed at necropsy, imaged for GFP expression as described above, and then whole mounted for morphological studies.

## Results and Discussion

### Imaging mammary ductal structure using reflectance confocal microscopy

**Figure 1:**
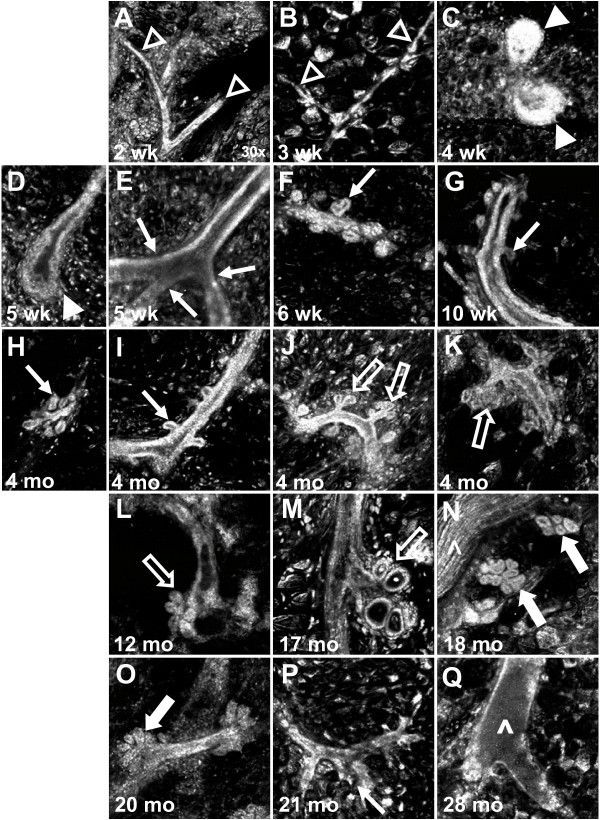
**Reflectance confocal microscopy (RCM) can visualize ductal epithelial cells during mammary gland development, growth, and aging**. Prominent features distinguishable with RCM are **(A-B) **rudimentary primary ductal trees (open arrowheads), **(C-D) **terminal end buds (closed arrowheads), **(E-I, P) **secondary branching (thin arrows), **(J-M) **tertiary branching (open arrows), **(N-O) **lobules (thick arrows), **(Q) **ductal ectasia or enlarged ducts (^). Magnification: 30×.

**Figure 2:**
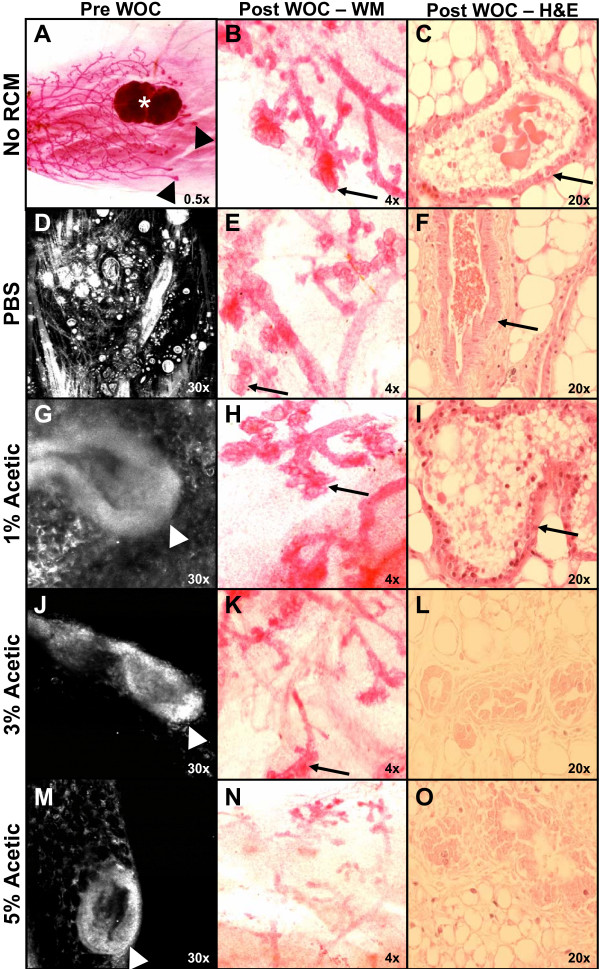
**RCM can image living tissue from mammary whole organ culture (WOC)**. Mammary glands from mice primed with an E&P pellet were either whole mounted immediately (uncultured) **(A) **or exposed to culture in IPAH supplemented media immediately **(B-C)**. Other glands were put into IPAH culture after RCM imaging which was accomplished by injection with either PBS **(D-F)**, 1% acetic acid **(G-I)**, 3% acetic acid **(J-L)**, or 5% acetic acid **(M-O)**. Pre-WOC RCM images **(D, G, J, M) **display examples of terminal end buds which can be compared to the whole mount (WM) image **(A)**. Alveolar development in the glands can be compared in post-WOC WM **(B, E, H, K, N) **and H&E **(C, F, I, L, O) **images. Arrowhead: terminal end bud, arrows: alveolar development, *: lymph node. Magnifications are as indicated.

Details of mammary ductal structure primary (Figure 1A and 1B), secondary (Figure 1E,F,G,H,I, and 1P) and tertiary branching (Figure 1J,K,L, and 1M), terminal end buds (Figure 1C and 1D), lobules (Figure 1N and 1O) and ductal ectasia (Figure 1Q) are readily visualized using reflectance confocal imaging. A 3-D rendering of terminal end bud structures is included as Additional File 1. To determine if this technique could be used to visualize structural elements prior to whole mammary organ culture, we treated mammary glands from E&P pellet primed five-week-old mice with PBS, 1%, 3%, or 5% acetic acid, imaged the gland with RCM, and then subjected them to WOC media supplemented with IPAH for 1 week. Quality of RCM images pre WOC (Figure 2 left column) were compared with the quality of whole mount (WM) and H&E obtained post WOC (Figure 2 middle and right columns). In the absence of acetic acid (PBS alone), ductal structures could not be distinguished in the RCM image (Figure 2D). One percent acetic acid was sufficient for visualization of terminal end bud structures, which were easily distinguishable with RCM because of their well-defined tear-drop shaped structure (Figure 2G) compared to non-imaged glands (Figure 2A), although image contrast, i.e. ability to distinguish individual cells in the multiple cell layers of the terminal end bud, was improved with 3% acetic acid (Figure 2J) and 5% acetic acid (Figure 2M). However, only the glands treated with 1% acetic acid were able to grow consistently and differentiate normally in response to IPAH in whole organ culture (Figure 2H and 2I) as compared to the non-imaged glands immediately put into culture (Figure 2B and 2C) and imaged glands treated with PBS alone (Figure 2E and 2F). Glands imaged with 3% acetic acid showed a partial viable response (Figure 2K and 2L), while no clear response was found in glands treated with 5% acetic acid (Figure 2N and 2O). In conclusion, RCM imaging using 1% acetic acid allows adequate visualization of ductal morphology while preserving the viability of the gland.

### Imaging alveolar, ductal, and tumor mammary development using GFP fluorescence

**Figure 3:**
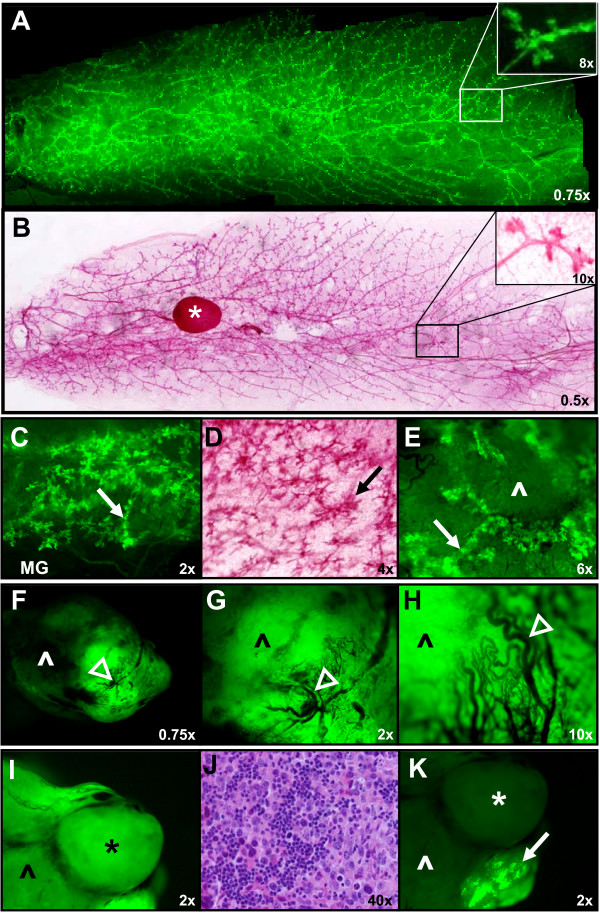
**GFP imaging can reveal details of ductal development, mammary tumors, tumor associated blood vessels, and lymph node morphology**. Ductal development can be compared with two imaging techniques – by first GFP imaging the mammary gland **(A, C) **and then whole mounting the same gland **(B, D)**. Mammary tumors appear less bright compared to the mammary ducts **(E) **and more bright compared to tumor associated blood vessels **(F-H)**. Lymph nodes **(I) **can be highlighted close to brighter mammary ducts **(K)**. **(J) **H&E section of a lymph node. Arrows: mammary ducts, ^: mammary tumor, open arrowheads: blood vessels, *: lymph node. Magnifications are as indicated.

GFP imaging of the whole mouse mammary gland (Figure 3A and 3C) allows for visualization with similar detail of ductal development as a whole mount (Figure 3B and 3D) even without the fat pad dissolution that occurs during whole mount fixation. Mammary tumors appear less bright when in the same field as brighter mammary ducts (Figure 3E) and homogeneously brighter when in the same field as tumor associated blood vessels which display reduced fluorescence (Figure 3F–H). Neovascularization of primary tumors can be imaged because the nonluminous angiogenic blood vessels are in sharp contrast to the brightly fluorescent tumor [24,48]. Lymph nodes (Figure 3I) can also be well appreciated when not in a field with brighter mammary ducts (Figure 3K) although not in as great detail as can be appreciated with the H&E section of a lymph node (Figure 3J).

**Figure 4:**
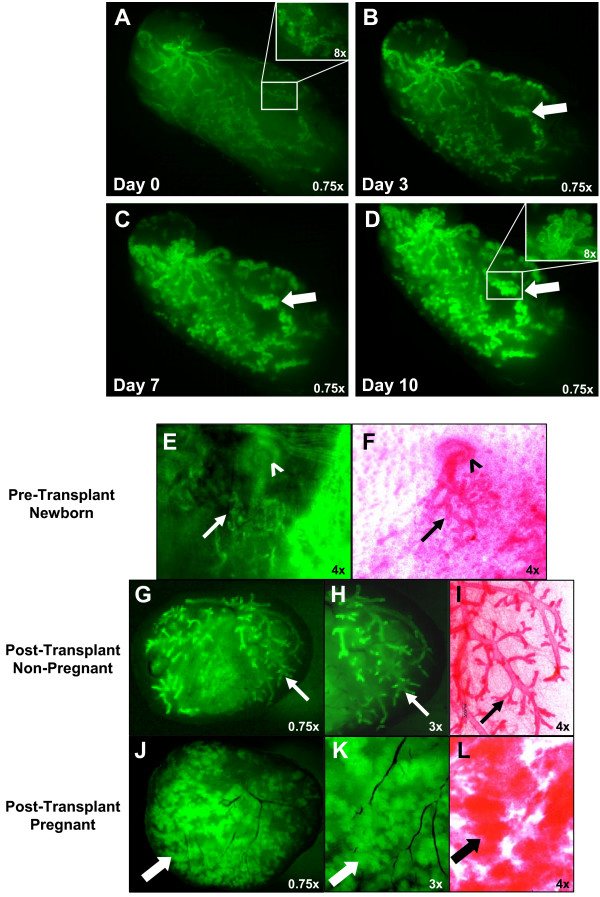
**GFP fluorescence monitors ductal and alveolar development in WOC and during mammary gland transplantation**. **(A-D) **Mammary glands were cultured in IPAH media for a total of 10 days. On days 0 **(A)**, 3 **(B)**, 7 **(C)**, and 10 **(D) **the mammary glands were briefly removed from the incubator to examine alveolar development as visualized with GFP expression. **(E-L) **Mammary glands from newborn mice expressing GFP were transplanted into nude mice. **E **and **F **show the newborn mammary gland pre-transplantation GFP and H&E imaging, respectively. Post-transplantation in a non-pregnant nude mouse **(G-I) **allows for visualization of ductal development of the transplanted gland as it grows to fill its fat pad whereas transplantation from a pregnant nude mouse **(J-L) **causes the transplant to display lobular development characteristic of late pregnancy. GFP images: **G **and **H **(higher power) and **J **and **K **(higher power), Whole mount images: **I **and **L**. Arrows: mammary ducts, thick arrows: lobules, ^: nipple. Magnifications are as indicated.

**Figure 5:**
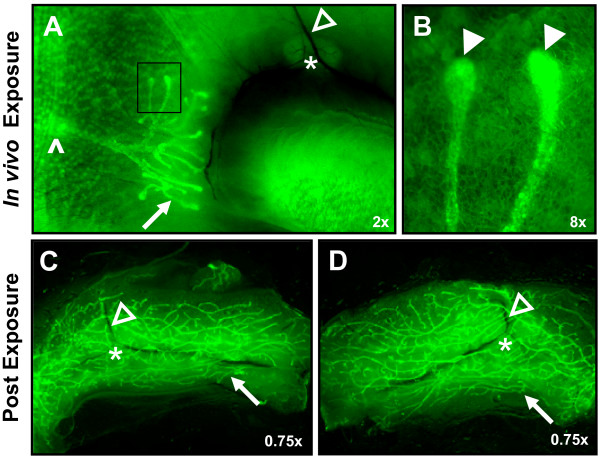
**Surgical exposure of the mammary gland for GFP imaging does not inhibit subsequent gland development**. A 4-week-old GFP mouse was anesthetized and one #4 mammary gland exposed *in vivo *and imaged. The epithelial tree is clearly visible, branching out from the nipple (^). **(A) **The lymph node (*) is visible at the junction of the blood vessels (open arrowheads). The dark crescent below the lymph node is caused by the mammary fat pad blocking the fluorescence of the underlying skin. **(B) **Two terminal end buds (marked with a box in panel **A**), at high magnification. After images were acquired the skin was closed and the mouse was allowed to recover. At 8 weeks of age the mouse was sacrificed and both #4 mammary glands were imaged for GFP fluorescence **(C-D)**. Both glands show a similar degree of mammary development. Arrows: mammary ducts, open arrowheads: blood vessels, closed arrowheads: terminal end buds, *: lymph node, ^: nipple. Magnifications are as indicated.

To establish whether the GFP imaging technique could be accomplished in conjunction with *in vivo *methods, we performed three mammary gland manipulations: whole organ culture, mammary gland transplantation, and *in vivo *surgical exposure. For the whole organ culture experiment, glands from GFP expressing mice were harvested and then imaged for Day 0 GFP fluorescence (Figure 4A). After imaging, the glands were exposed to IPAH media in culture and then imaged periodically for a total of 10 days (Figure 4B–D). Increasing amounts of GFP fluorescence shows that the alveolar development in response to the IPAH stimulation can be followed *in vivo*. Similarly, mammary glands from newborn GFP expressing mice (Figure 4E and 4F) were transplanted into nude mice.  GFP expression was appreciated in the resulting mammary ductal development in transplants from non-pregnant host nude mice (Figure 4G and 4H) and in the lobular development in transplants from pregnant host nude mice (Figure 4J and 4K).  Both compare well to non-pregnant and pregnant whole mounts (Figure 4I and 4L, respectively). For the *in vivo *exposure experiment, a 4-week-old GFP expressing mouse was anesthetized and one #4 mammary gland exposed *in vivo *and imaged. The lymph node, mammary ducts extending from the nipple, and terminal end buds were clearly visible by GFP fluorescence (Figure 5A and 5B). After imaging, the mouse was allowed to recover. Both #4 mammary glands were dissected 4 weeks after *in vivo *exposure and showed a similar degree of mammary development by GFP imaging (Figure 5C and 5D) indicating that surgical exposure and imaging of the mammary gland did not alter its development.

### Imaging non-palpable mammary gland lesions using ultrasound

**Figure 6:**
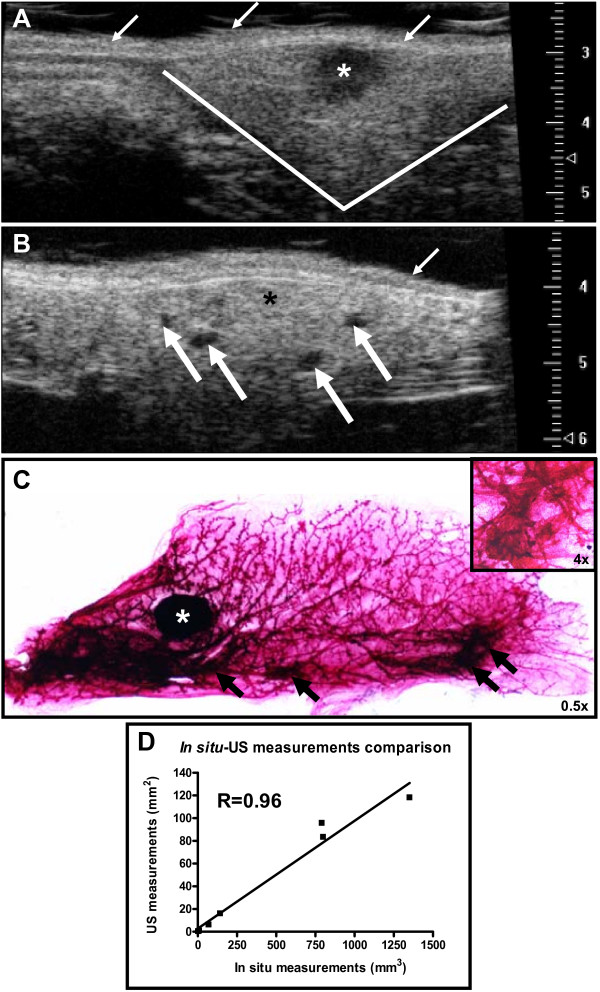
**Ultrasound can detect nonpalpable lesions in the mammary gland of transgenic mice**. **(A) **Fourth mammary gland ultrasound image from a control wild-type mouse illustrating the homogeneous texture of the mammary gland tissue that surrounds the centrally located lymph node (*) with lines indicating the border of the #4 mammary gland. **(B) **Ultrasound image of the left #4 mammary gland of a tTA/TAg/ERα mouse with nonpalpable mammary lesions (thick arrows). The dense (less echogenic) tissue represents an area of increased cell number in the tTA/TAg/ERα mammary gland and correlates with the presence of hyperplastic alveolar nodules (HANs) identified after carmine-alum whole mount staining of the same mammary gland at necropsy **(C)**. Image: 0.5×, insert: 4×. Thick arrows: HANs, white (ultrasound) or black (whole mount), thin white arrows: skin surface, *: lymph node. Scale for all ultrasound images: in mm. **(D) **Correlation between 2-D ultrasound and 3-D *in situ *measurements of mammary preneoplasias (n = 7 mice); R = 0.96, p < 0.001.

**Figure 7:**
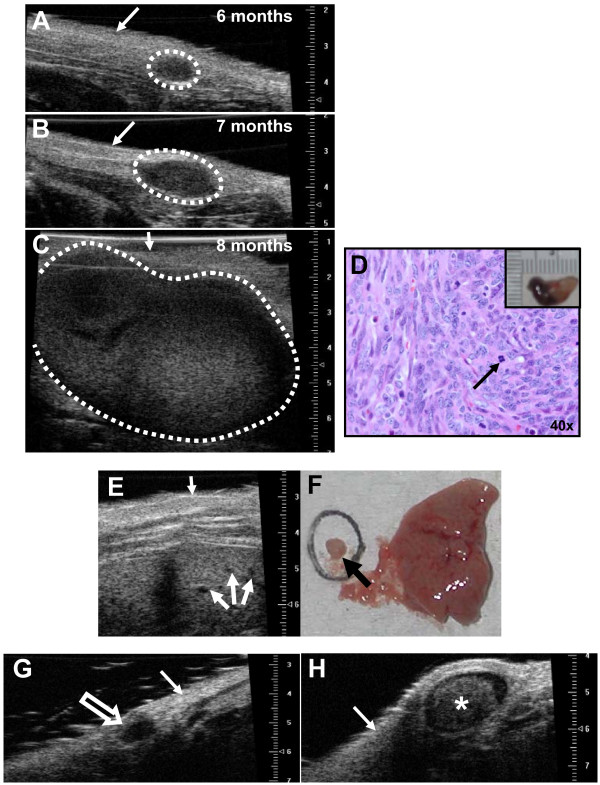
**Ultrasound can distinguish between different lesions in transgenic mice *in vivo***. Possible applications are to identify and follow mammary tumor development and progression over time **(A-C) **and compare to the appearance of tumor at time of necropsy **(D)**, identify liver metastasis **(E-F)**, distinguish between adenocarcinoma **(G) **and enlarged lymphnodes **(H)**. Thin white arrow: skin surface, thin black arrow: mitotic figure, thick white arrows: liver metastases, circle and black arrow: dissected metastatic lesion, open white arrow: tumor, *: enlarged lymph node. Scale for all ultrasound images in mm.

Normal mammary gland demonstrates a relatively homogenous echogenic imaging pattern (Figure 6A) with ultrasound. Lymph nodes (*) within the gland can be distinguished from surrounding gland structure. Similarly, non-palpable preneoplastic mammary lesions can be visualized and distinguished from normal gland and lymph node structure and size can be measured using ultrasound imaging (Figure 6B). The less echogenic (dark spots, arrows) preneoplastic lesions correlate well with hyperplastic alveolar nodules (HANs) observed on whole mount (Figure 6C–D). While detailed ductal and cellular structure cannot be appreciated with ultrasound, preneoplastic lesions can be followed over time to establish growth rates of mammary tumor development (Figure 7A–D). Serial ultrasound was performed to image the growth of a mammary adenocarcinoma over time from a tTA/TAg/ERα mouse at 6 **(A)**, 7 **(B)**, and 8 **(C) **months of age as compared to the appearance of the tumor at the time of necropsy **(D). **Using ultrasonography we were able to detect mammary preneoplastic lesions as small as 0.2 mm (with an area of 0.09 mm^2^) at least 1.5 to 2 months prior to becoming palpable. In addition, multiple liver metastases from an 11-month-old tTA/TAg/ERα mouse with a mammary adenocarcinoma were visualized with ultrasound (Figure 7E) and compared to the metastatic lesion dissected out from the liver (Figure 7F). Ultrasound also proved useful in identifying a 1-mm^3 ^solid mammary adenocarcinoma from a tTA/TAg/ERα mouse (Figure 7G). Note the absence of internal echoes consistent with relatively homogeneous tissue. Ultrasound can also distinguish between an adenocarcinoma and an enlarged (1.5- to 2-mm^3^) lymph node (Figure 7H) which was adjacent to the #2 mammary gland of a tTA/TAg/ERα mouse and was confirmed as a lymph node at necropsy.

## Conclusion

The studies presented here compare the well-established techniques of mammary gland whole mounting and hematoxylin and eosin histology with RCM, GFP, and ultrasound to study mammary gland and mammary tumor development. RCM, GFP, and ultrasound are quick techniques that do not require tissue processing for immediate imaging of mammary gland structures. RCM has the potential to advance screening and diagnosis, especially for the early detection of a variety of precancerous lesions [13,49-51]. We have shown here and previously that RCM is very useful in optical serial imaging of normal mammary gland ductal structures and tumors in harvested tissues from genetically engineered mice [15]. It has a resolution comparable to the ductal structure resolution of a mammary whole mount and the cellular resolution of mammary histology. This technique can be used for 3-D reconstruction of MG morphology and can be used in living tissue. In this study RCM was also used in combination with whole mammary gland organ culture. Additional RCM applications include using it to excise specific mammary structures for transplant studies. GFP imaging is also useful for *in vivo *studies, as well as for whole organ culture and transplantation, where it can be used to follow development and/or disease progression. GFP has been shown to be invaluable in mammary gland transplantation studies where it can successfully answer such questions as whether a growth factor acts in an autocrine or paracrine fashion during mammary gland development by allowing for labeling and following the development of specific mammary cells transplanted into a fat pad [52]. Ultrasound demonstrates the least cellular resolution and requires an experienced operator to obtain consistent images, but is very useful for *in vivo *and non-invasive imaging of development of non-palpable preneoplastic lesions into mammary adenocarcinomas. 3-D ultrasound imaging software can be used to obtain direct measurements of lesion volume, if needed. In summary, all three techniques are valuable adjuvants to the study of mammary development and cancer progression.

The imaging modalities used in this paper, RCM, GFP, and ultrasound imaging, are just a few of the many techniques being developed to study the mammary gland and mammary cancer. These versatile techniques can be combined with each other (i.e. fluorescence and RCM), as well as with other techniques, such as those that involve the detection of specific probes to image targeted cells while simultaneously acquiring confocal contrast images to localize the targeted cells within the histological context of the tissue being imaged [53,54]. Combining these techniques allows the researcher to obtain actual *in vivo *molecular expression information from the image enabling the study of the molecular basis of initiation and progression of mammary cancer. All of these minimally invasive techniques allow longitudinal imaging to provide complete and precise information about mammary gland development, as well as, tumor initiation and progression in any transgenic cancer mouse model. The further development of mouse imaging techniques may well lead to the advancement of new technologies that can be translated to more sensitively and specifically detect precancerous abnormalities, diagnose curable pre-cancerous lesions, and to increase patient survival and quality of life in breast cancer patients.

## Abbreviations

2-D, two-dimensional; 3-D, three-dimensional; CMV, cytomegalovirus; E&P pellet, 17β-estradiol + progesterone pellet; ERα, Estrogen Receptor alpha; GFP, green fluorescent protein; H&E, Hematoxylin and Eosin; LN, lymph node; IPAH media, insulin + prolactin + aldosterone + hydrocortisone supplemented media; PBS, phosphate buffered saline; RCM, Reflectance Confocal Microscopy; WM, Whole Mount; WOC, Whole Organ Culture.

## Competing interests

The author(s) declare that they have no competing interests.

## Authors' contributions

MTT performed the RCM whole organ culture experiment, comparison of GFP and whole mount, GFP lymphnode imaging, supervised and performed the ultrasound to follow preneoplasia and tumor progression experiments, and wrote and coordinated the manuscript. ARP performed the RCM of mice during development and during the whole organ culture experiment. IC performed the GFP mammary gland transplant experiment and the comparison of ultrasound and *in situ *measurements. LPJ performed the GFP whole organ culture experiment. MDJ initially developed the GFP imaging procedures, performed the live GFP imaging, and supervised imaging of whole mammary gland organ cultures, mammary gland transplants, and the mouse mammary tumor. PAF designed and supervised the experiments in collaboration with the other investigators and edited the manuscript. All authors have read and approved the final version of the manuscript.

## Pre-publication history

The pre-publication history for this paper can be accessed here:



## Supplementary Material

Additional file 1 **3-D rendering of mammary gland terminal end buds**. The VivaCell 5000 software acquires multiple high resolution digital image slices vertically through mammary gland morphological structures, such as the terminal end bud, called VivaStacks. The video is a compilation of three VivaStacks, each consisting of 16 images at 1.585 μm increments, which included entire terminal end bud structures. The Volume Viewer plug-in of the ImageJ software (Image Analysis and Processing in Java, the National Institutes of Health, Bethesda, MD) [55] artificially colored the images from the combination of all the stacks and produced a 3-D representation. Click here for file
